# Effect of obesity on human reproduction

**DOI:** 10.1186/1751-0147-57-S1-K3

**Published:** 2015-09-25

**Authors:** Inger Sundström Poromaa

**Affiliations:** 1 Department of Women's and Children's Health, Uppsala University, Uppsala, Sweden

## 

Obesity influences human reproduction in several ways. Obesity is associated with sub-fertility and this effect is noticeable already at BMI 25 kg/m^2^. As many as 6 - 9% of the female population of child-bearing age suffer from polycystic ovary syndrome, i.e. anovulatory infertility, among whom more than half are obese. Bariatric surgery has become increasingly common in women of childbearing ages, and poses specific problem when women later become pregnant. In addition, the obesity epidemic has also reached the pregnant population of developed countries. The challenge for clinicians within the obstetric field is three-fold; first, obesity is associated with a number of obstetric risks that may have fatal or long-term consequences for both mother and offspring such as preeclampsia, preterm birth, intrauterine death, and congenital malformations. Secondly, while the obvious cure for obesity is weight loss, this is not an appropriate strategy during pregnancy when fetal nutritional demands are at stake. Finally, Caesarean section delivery is not the answer to the problem; the adverse neonatal outcomes are unaffected by mode of delivery, and the anesthetic risks, that are generally elevated in pregnant women, are even more imminent in obese pregnant women.

